# Empagliflozin after myocardial infarction with or without diabetes and chronic kidney disease: Insights from EMPACT‐MI

**DOI:** 10.1002/ehf2.15393

**Published:** 2025-09-14

**Authors:** Francesco Fioretti, Javed Butler, Jacob A. Udell, W. Schuyler Jones, Mark C. Petrie, Josephine Harrington, Michaela Mattheus, Johann Bauersachs, Antoni Bayes‐Genis, Shaun G. Goodman, Tomasz Gasior, James L. Januzzi, Renato D. Lopes, Piotr Ponikowski, Xavier Rossello, Morten Schou, Peter van der Meer, Dragos Vinereanu, Shelley Zieroth, Martina Brueckmann, Mikhail Sumin, Deepak L. Bhatt, Adrian F. Hernandez, Stefan D. Anker

**Affiliations:** ^1^ Baylor Scott & White Research Institute Dallas Texas USA; ^2^ Cardiology Unit ASST Spedali Civili Hospital and University of Brescia Brescia Italy; ^3^ Women's College Hospital and Peter Munk Cardiac Centre Toronto General Hospital, University of Toronto Toronto Canada; ^4^ Division of Cardiology, Department of Medicine and Duke Clinical Research Institute Duke University School of Medicine Durham North Carolina USA; ^5^ School of Cardiovascular and Medical Sciences, British Heart Foundation Glasgow Cardiovascular Research Centre University of Glasgow Glasgow UK; ^6^ Boehringer Ingelheim Pharma GmbH & Co. KG Ingelheim Germany; ^7^ Department of Cardiology and Angiology Hannover Medical School Hanover Germany; ^8^ Heart Institute Hospital Universitari Germans Trias i Pujol Barcelona Spain; ^9^ Department of Medicine Universitat Autònoma de Barcelona Barcelona Spain; ^10^ Canadian VIGOUR Centre University of Alberta Edmonton Canada; ^11^ Division of Cardiology, Department of Medicine, St. Michael's Hospital, Unity Health Toronto and Peter Munk Cardiac Centre University Health Network, University of Toronto Toronto Canada; ^12^ Boehringer Ingelheim International GmbH Ingelheim Germany; ^13^ Collegium Medicum—Faculty of Medicine WSB University Dąbrowa Górnicza Poland; ^14^ Division of Cardiology Harvard Medical School and Massachusetts General Hospital, Baim Institute for Clinical Research Boston Massachusetts USA; ^15^ Institute for Heart Diseases Wrocław Medical University Wrocław Poland; ^16^ Hospital Universitari Son Espases, Health Research Institute of the Balearic Islands University of the Balearic Islands Palma de Mallorca Spain; ^17^ Department of Cardiology Herlev and Gentofte University Hospital Copenhagen Denmark; ^18^ Department of Cardiology University of Groningen, University Medical Center Groningen Groningen The Netherlands; ^19^ University of Medicine and Pharmacy Carol Davila, University and Emergency Hospital Bucharest Romania; ^20^ Section of Cardiology, Max Rady College of Medicine University of Manitoba Winnipeg Canada; ^21^ First Department of Medicine, Medical Faculty Mannheim Heidelberg University Mannheim Germany; ^22^ Mount Sinai Fuster Heart Hospital, Icahn School of Medicine at Mount Sinai New York New York USA; ^23^ Department of Cardiology (CVK) German Heart Center Charité, German Centre for Cardiovascular Research (DZHK) Partner Site Berlin, Charité—Universitätsmedizin Berlin Berlin Germany

**Keywords:** CKD, diabetes, heart failure, myocardial infarction, recommendation, SGLT2i

## Abstract

**Background:**

In the EMPACT‐MI trial, empagliflozin did not reduce the primary endpoint of all‐cause mortality or hospitalization for heart failure (HHF) following acute myocardial infarction (AMI) but was associated with a risk reduction for HF events.

**Objectives:**

This study aimed to evaluate whether the effect of empagliflozin on HF events is consistent in patients with and without type 2 diabetes and/or chronic kidney disease enrolled in the EMPACT‐MI trial.

**Methods:**

Post hoc analysis assessing the effect of empagliflozin on the primary endpoint and on HF events in AMI patients with and without an established recommendation for a sodium–glucose cotransporter‐2 inhibitor (SGLT2i) (type 2 diabetes or chronic kidney disease).

**Results:**

Of 6522 participants, 3489 (53%) did not have type 2 diabetes and/or chronic kidney disease. Those without these conditions were younger and with fewer comorbidities. No differences were observed for the primary endpoint. Empagliflozin reduced time to first HHF, total HHF, time to adverse event (AE) of HF (including outpatient HF events) and total AEs of HF similarly in patients with and without type 2 diabetes or chronic kidney disease. Total HHFs were 50 and 63 [adjusted event rate 1.74 and 2.31 events per 100 patient‐years; rate ratio (RR) 0.75; 95% confidence interval (CI) 0.48, 1.18] in patients without and 98 and 144 (adjusted event rate 3.91 and 6.04 events per 100 patient‐years; RR 0.65; 95% CI 0.45, 0.94; *P* for interaction = 0.61) in those with type 2 diabetes or chronic kidney disease in the empagliflozin and placebo arms, respectively. Any AEs, serious AEs and AEs leading to permanent study drug discontinuation were similar between treatment groups in both subgroups.

**Conclusions:**

Empagliflozin improved HF outcomes similarly in patients after AMI with or without type 2 diabetes or chronic kidney disease.

## Graphical abstract

Impact of empagliflozin in patients with and without T2D or CKD in the EMPACT‐MI trial.

Baseline characteristics, HF outcomes and subgroup analysis of efficacy endpoints are shown.

^#^ Hazard ratios estimated via Cox proportional hazard model (unless indicated otherwise).

^ Rate ratios via negative binomial regression.

ACM = all‐cause mortality, AE = adverse event, BMI = body mass index, eGFR = estimated glomerular filtration rate, HHF = hospitalization for heart failure, SGLT2 = sodium glucose cotransporter‐2.

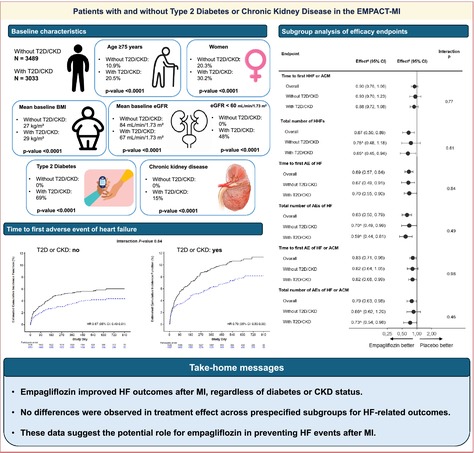

## Introduction

New onset of heart failure (HF) after acute myocardial infarction (AMI) is associated with worse outcomes.^1^, ^2^, ^3^, ^4^, ^5^, ^6^ Despite advances in interventional and drug therapies targeting recurrent myocardial infarction (MI), no new therapy has been discovered to reduce the risk of new‐onset HF after AMI in two decades. Sodium–glucose cotransporter‐2 inhibitors (SGLT2is) have been consistently shown to reduce the risk of HF events in patients at high risk of developing HF, including those with type 2 diabetes (T2D) and chronic kidney disease (CKD), and in patients with prevalent HF, irrespective of left ventricular (LV) ejection fraction (LVEF).^7^, ^8^, ^9^ In the Effect of Empagliflozin on Hospitalization for Heart Failure and Mortality in Patients With Acute Myocardial Infarction (EMPACT‐MI) trial, although empagliflozin did not reduce the primary endpoint of time to first hospitalization for HF (HHF) or all‐cause death, it had an effect on HF outcomes, with a relative risk reduction of 23% for time to first HHF and 33% for total (first and recurrent) HHF.^10^, ^11^

Current guideline recommendations for the use of SGLT2i include patients with HF, T2D and CKD.^12^, ^13^ While patients with prevalent HF were excluded from the EMPACT‐MI, those with T2D and CKD who have an established recommendation for an SGLT2i are at higher risk for adverse outcomes after AMI and were included. Whether empagliflozin has a favourable impact on HF outcomes in post‐AMI patients without T2D and/or CKD is unknown. In this post hoc analysis of the EMPACT‐MI trial, we aimed to compare the effect of empagliflozin on HF outcomes in patients with or without an established recommendation for an SGLT2i.

## Methods

### Study design and patient population

The study design and results of EMPACT‐MI have been published.^11^, ^14^ In brief, EMPACT‐MI was a randomized, parallel‐group, double‐blind, placebo‐controlled, event‐driven trial. The trial was approved by the ethics committee at each site, and all patients provided written informed consent. EMPACT‐MI enrolled patients 18 years of age or older, hospitalized for ST‐segment elevation MI (STEMI) or non‐STEMI (NSTEMI) within 14 days of admission. Each patient had either evidence of newly developed LVEF < 45% or signs or symptoms of congestion requiring treatment during index hospitalization. In addition, patients were required to have at least one enrichment factor, including ≥65 years of age; new LVEF < 35%; history of MI; atrial fibrillation; T2D; estimated glomerular filtration rate (eGFR) <60 mL/min/1.73 m^2^; elevated natriuretic peptides (NPs) or uric acid levels; elevated pulmonary artery or right ventricular systolic pressure; three‐vessel coronary artery disease; peripheral artery disease; or no revascularization for index MI. Patients with a diagnosis of HF prior to index MI who were taking or planned the use of SGLT2i, had an eGFR < 20 mL/min/1.73 m^2^ or were on dialysis were excluded. Patients were randomly assigned to receive either placebo or 10 mg of empagliflozin daily in a 1:1 ratio, in addition to standard of care. In the current analysis, T2D was defined as a previous history of T2D as assessed at baseline, whereas CKD was defined as a history of CKD or eGFR < 60 mL/min/1.73 m^2^ at baseline.

### Outcomes

The primary endpoint of EMPACT‐MI was a composite of time to first HHF or all‐cause death.^11^ The key secondary outcomes included time to first HHF, time to all‐cause death and total number (first and recurrent) of HHFs.^11^ In this post hoc analysis, we also examined investigator‐reported adverse events (AEs) that were categorized as ‘cardiac failure’ per Medical Dictionary for Regulatory Activities (MedDRA) standards. They included not only the events analysed as the pre‐specified endpoint of HHF but also a broader range of AEs of HF, including outpatient nonfatal HF AEs as well as those requiring or prolonging hospitalization or with a fatal outcome. Thus, we also analysed time to first AE of HF, total number of AEs of HF, time to first AE of HF or all‐cause death, and total number of AEs of HF or all‐cause mortality. A similar approach was used previously.^10^

### Statistical analysis

Baseline characteristics of study participants with and without T2D and/or CKD were evaluated. Comparison between the empagliflozin and placebo arms for time to first event endpoints was performed using the primary Cox proportional hazard model, including baseline covariates of age, geographical region, eGFR [assessed categorically using the Chronic Kidney Disease Epidemiology Collaboration (CKD‐EPI) formula <45 vs. 45 to <60 vs. 60 to <90 vs. ≥90 mL/min/1.73 m^2^], LVEF (<35% vs. ≥35%), T2D, atrial fibrillation, previous MI, peripheral arterial disease, smoking status and the additional covariate of sex. Data for patients who did not have an event were censored on the last day they were known to be free of the outcome. The assumption of proportional hazards was verified.^15^ For comparison of total (first and recurrent) events, differences between empagliflozin and placebo were assessed using a negative binomial model including the same covariates and including logarithm of time as an adjustment for observation time.^16^

Consistency of the effect of empagliflozin on all endpoints evaluated in this analysis was assessed across two groups: patients with an established recommendation (T2D and/or CKD) and those without an established recommendation for an SGLT2i. These analyses were performed based on the Cox regression and negative binomial regression models including factors as described previously (excluding eGFR and T2D as separate factors) and additional terms for subgroup (i.e., combination of eGFR and T2D categories) and interaction of subgroup by treatment (with interaction tests). All *P* values reported for these exploratory analyses are two‐sided, and a *P* value <0.05 was considered statistically significant.

## Results

### Baseline characteristics of the overall population

From December 2020 to March 2023, 6522 study participants were randomized to empagliflozin (*n* = 3260) or placebo (*n* = 3262). Of these, 3489 (53%) did not and 3033 (47%) did have CKD or T2D. Baseline characteristics of patients are shown in the Graphical Abstract and *Tables* *1*, S1 and S2.

**Table 1:** Baseline characteristics of patients with and without T2D or CKD enrolled in the EMPACT‐MI trial.

	Patients without baseline eGFR < 60 mL/min/1.73 m^2^ and history of CKD or baseline T2D (*n* = 3489)			Patients with baseline eGFR < 60 mL/min/1.73 m^2^ and history of CKD or baseline T2D (*n* = 3033)			*P* value^c^
	Empagliflozin *n* = 1753	Placebo *n* = 1736	Total *n* = 3489	Empagliflozin *n* = 1507	Placebo *n* = 1526	Total *n* = 3033	
Age (years)	61.7 ± 11.0	61.8 ± 10.8	61.8 ± 10.9	65.8 ± 10.6	65.7 ± 10.5	65.8 ± 10.5	<0.0001
Age ≥75 years, no. (%)	202 (11.5)	179 (10.3)	381 (10.9)	317 (21.0)	305 (20.0)	622 (20.5)	<0.0001
Women, no. (%)	349 (19.9)	359 (20.7)	708 (20.3)	463 (30.7)	454 (29.8)	917 (30.2)	<0.0001
Region, no. (%)							
North America	252 (14.4)	238 (13.7)	490 (14.0)	179 (11.9)	195 (12.8)	374 (12.3)	0.0008
Latin America	134 (7.6)	133 (7.7)	267 (7.7)	156 (10.4)	155 (10.2)	311 (10.3)	
Europe	1164 (66.4)	1163 (67.0)	2327 (66.7)	989 (65.6)	991 (64.9)	1980 (65.3)	
Asia	203 (11.6)	202 (11.6)	405 (11.6)	183 (12.1)	185 (12.1)	368 (12.1)	
Race^a^, no. (%)							
White	1457 (83.1)	1450 (83.5)	2907 (83.3)	1273 (84.5)	1271 (83.3)	2544 (83.9)	0.50
Black/African American	27 (1.5)	25 (1.4)	52 (1.5)	17 (1.1)	23 (1.5)	40 (1.3)	
Asian	223 (12.7)	212 (12.2)	435 (12.5)	198 (13.1)	201 (13.2)	399 (13.2)	
Other including mixed race	9 (0.5)	2 (0.1)	11 (0.3)	0	5 (0.3)	5 (0.2)	
Smoking status, no. (%)							
Current	710 (40.5)	670 (38.6)	1380 (39.6)	418 (27.7)	421 (27.6)	839 (27.7)	<0.0001
Non‐current (former/never)	1043 (59.5)	1066 (61.4)	2109 (60.4)	1089 (72.3)	1105 (72.4)	2194 (72.3)	
Time from index admission to discharge (days)	6.06 ± 4.74	6.16 ± 4.62	6.11 ± 4.68	6.91 ± 9.16	6.71 ± 6.00	6.81 ± 7.73	<0.0001
Time from index admission to study drug intake (days)	5.60 ± 3.46	5.53 ± 3.43	5.57 ± 3.44	5.96 ± 3.48	5.75 ± 3.46	5.85 ± 3.47	0.0009
Cardiovascular history, no. (%)							
Prior MI	172 (9.8)	219 (12.6)	391 (11.2)	216 (14.3)	240 (15.7)	456 (15.0)	<0.0001
Prior stroke/TIA	58 (3.3)	59 (3.4)	117 (3.4)	80 (5.3)	105 (6.9)	185 (6.1)	<0.0001
Atrial fibrillation	168 (9.6)	151 (8.7)	319 (9.1)	190 (12.6)	210 (13.8)	400 (13.2)	<0.0001
Type 2 diabetes	0	0	0	1035 (68.7)	1046 (68.5)	2081 (68.6)	
Chronic kidney disease	0	0	0	221 (14.7)	233 (15.3)	454 (15.0)	
Peripheral artery disease	95 (5.4)	97 (5.6)	192 (5.5)	77 (5.1)	83 (5.4)	160 (5.3)	0.68
Arterial hypertension	1049 (59.8)	1047 (60.3)	2096 (60.1)	1213 (80.5)	1229 (80.5)	2442 (80.5)	<0.0001
Key cardiovascular medications at baseline, no. (%)							
ACEis/ARBs	1199 (68.4)	1187 (68.4)	2386 (68.4)	1056 (70.1)	996 (65.3)	2052 (67.7)	0.83
ARNI	81 (4.6)	83 (4.8)	164 (4.7)	77 (5.1)	88 (5.8)	165 (5.4)	0.17
Beta‐blockers	1339 (76.4)	1344 (77.4)	2683 (76.9)	1176 (78.0)	1190 (78.0)	2366 (78.0)	0.29
Diuretics	912 (52.0)	903 (52.0)	1815 (52.0)	911 (60.5)	950 (62.3)	1861 (61.4)	<0.0001
MRAs	681 (38.8)	669 (38.5)	1350 (38.7)	587 (39.0)	636 (41.7)	1223 (40.3)	0.18
Loop diuretics	493 (28.1)	469 (27.0)	962 (27.6)	641 (42.5)	609 (39.9)	1250 (41.2)	<0.0001
Thiazides	73 (4.2)	84 (4.8)	157 (4.5)	106 (7.0)	103 (6.7)	209 (6.9)	<0.0001
Statins	1574 (89.8)	1542 (88.8)	3116 (89.3)	1333 (88.5)	1349 (88.4)	2682 (88.4)	0.26
Anti‐thrombotic drugs	1672 (95.4)	1633 (94.1)	3305 (94.7)	1425 (94.6)	1443 (94.6)	2868 (94.6)	0.77
Baseline SBP (mmHg)	118.7 ± 14.2	119.2 ± 14.9	118.9 ± 14.5	122.1 ± 14.8	122.0 ± 15.3	122.1 ± 15.1	<0.0001
Baseline heart rate (b.p.m.)	72.6 ± 11.2	73.1 ± 11.6	72.8 ± 11.4	73.9 ± 10.9	74.4 ± 11.4	74.2 ± 11.1	<0.0001
Baseline BMI (kg/m^2^)	27.38 ± 4.77	27.38 ± 4.82	27.38 ± 4.79	28.89 ± 5.16	28.84 ± 5.15	28.87 ± 5.15	<0.0001
Baseline serum creatinine (mg/dL)	0.9 ± 0.2	0.9 ± 0.2	0.9 ± 0.2	1.1 ± 0.3	1.1 ± 0.4	1.1 ± 0.4	<0.0001
Baseline eGFR (CKD‐EPI) (mL/min/1.73 m^2^)	84.08 ± 13.88	84.06 ± 13.52	84.07 ± 13.70	67.14 ± 21.89	66.83 ± 22.07	66.98 ± 21.98	<0.0001
Baseline eGFR < 60 mL/min/1.73 m^2^, no. (%)	0	0	0	720 (47.8)	738 (48.4)	1458 (48.1)	
Baseline NT‐proBNP (pg/mL)	2583.43 ± 3439.64	2490.62 ± 2861.27	2534.71 ± 3148.52	3510.79 ± 4790.80	3847.68 ± 5229.74	3673.45 ± 5008.09	0.0001^d^
Baseline LVEF^a^, no. (%)							
LVEF < 35%	413 (23.6)	376 (21.7)	789 (22.6)	292 (19.4)	312 (20.4)	604 (19.9)	0.008
LVEF ≥ 35%	1327 (75.7)	1346 (77.5)	2673 (76.6)	1204 (79.9)	1200 (78.6)	2404 (79.3)	
Low‐risk profile^a^ ^,^ ^b^, no. (%)	594 (33.9)	655 (37.7)	1249 (35.8)	437 (29.0)	399 (26.1)	836 (27.6)	<0.0001
Number of enrichment criteria ≥4, no. (%)	117 (6.7)	123 (7.1)	240 (6.9)	583 (38.7)	599 (39.3)	1182 (39.0)	<0.0001

Abbreviations: ACEis, angiotensin‐converting enzyme inhibitors; ARBs, angiotensin II receptor blockers; ARNI, angiotensin receptor/neprilysin inhibitor; BMI, body mass index; CKD, chronic kidney disease; CKD‐EPI, Chronic Kidney Disease Epidemiology Collaboration; eGFR, estimated glomerular filtration rate; LVEF, left ventricular ejection fraction; MI, myocardial infarction; MRAs, mineralocorticoid receptor antagonists; NT‐proBNP, N‐terminal pro‐B type natriuretic peptide; SBP, systolic blood pressure; T2D, type 2 diabetes; TIA, transient ischaemic attack.

^a^ Number of patients with missing data for race: in ‘subgroup without’ *N* = 37 in empagliflozin and *N* = 47 in placebo and in ‘subgroup with’ *N* = 19 in empagliflozin and *N* = 26 in placebo; for time from index MI diagnosis to randomization: in ‘subgroup without’ *N* = 1 in empagliflozin and *N* = 0 in placebo and in ‘subgroup with’ *N* = 1 in empagliflozin and *N* = 0 in placebo; for baseline LVEF: in ‘subgroup without’ *N* = 13 in empagliflozin and *N* = 14 in placebo and in ‘subgroup with’ *N* = 11 in empagliflozin and *N* = 14 in placebo; and for low‐risk profile: in ‘subgroup without’ *N* = 0 in empagliflozin and *N* = 0 in placebo and in ‘subgroup with’ *N* = 1 in empagliflozin and *N* = 0 in placebo.

^b^ Low‐risk profile: if the patient had lowest LVEF ≥ 35% and no signs/symptoms of congestion requiring treatment.

^c^ Analysis conducted with pooled empagliflozin and placebo; *t* test for continuous variables and chi‐squared test for categorical variables.

^d^ Based on log‐transformed results.

### Characteristics and treatment of patients with and without T2D or CKD

Main baseline characteristics are summarized in the Graphical Abstract. Study participants without T2D or CKD were younger (62 ± 11 vs. 66 ± 11 years; *P* < 0.0001), were more likely male (79.7% vs. 69.8%; *P* < 0.0001), were more often current smokers (39.6% vs. 27.7%; *P* < 0.0001), had fewer comorbidities, had less loop diuretic use (27.6% vs. 41.2%; *P* < 0.0001), and were more likely to have baseline LVEF < 35% (22.6% vs. 19.9%; *P* = 0.008) (*Table* *1*). In these patients, STEMI was more common (78.1% vs. 70.0%; *P* < 0.0001), and signs or symptoms of congestion were less frequent (58.3% vs. 69.6%; *P* < 0.0001) (Table S1). In both groups, revascularization was done in most cases (91.4% in patients without vs. 86.8% in those with T2D or CKD; *P* < 0.0001). Enrichment criteria differed between the two groups, including age ≥65 years (43.9% vs. 56.9%; *P* < 0.0001), prior MI (11.2% vs. 15%; *P* < 0.0001), atrial fibrillation (8.8% vs. 12.6%; *P* < 0.0001), no revascularization for index AMI (6.6% vs. 10.1%; *P* < 0.0001) and three‐vessel coronary disease (30.0% vs. 32.2%; *P* = 0.0002), being more common in those with T2D or CKD (Table S2). Among those enrichment criteria reported, only 6.9% of study participants without and 39.0% with T2D or CKD had ≥4 enrichment criteria (*Table* *1*).

### Event rates in the placebo arm

**Figure 1:**
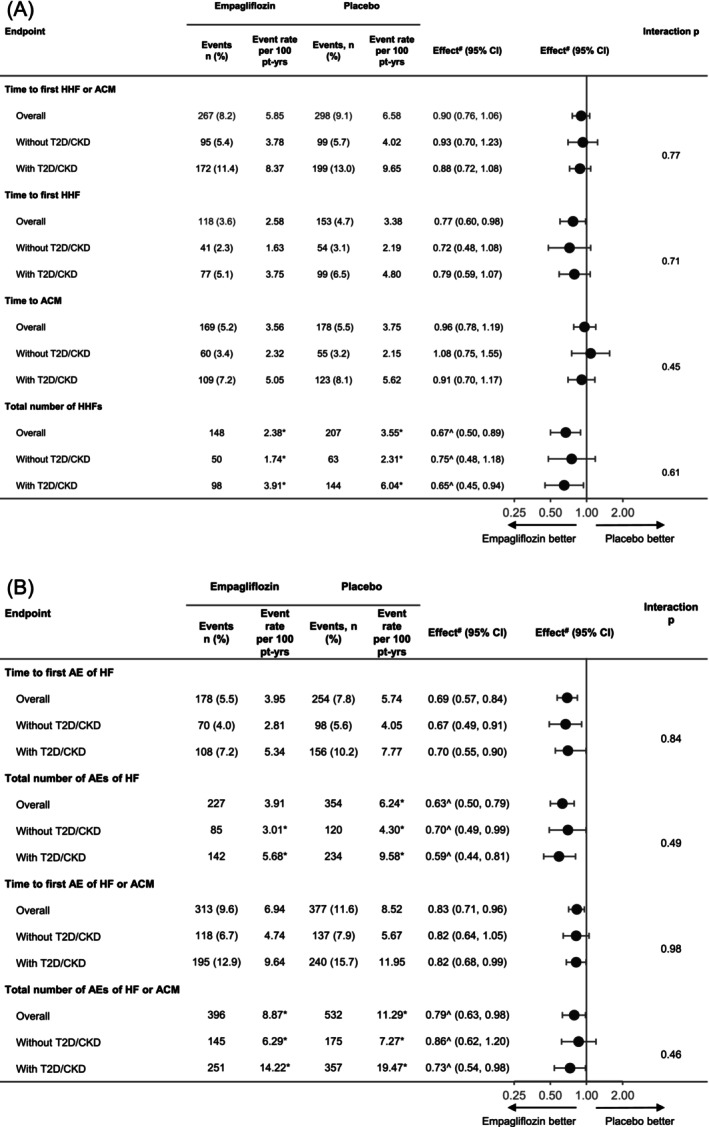
Subgroup analysis of efficacy endpoints. (A) Hospitalization for heart failure (HHF) and all‐cause mortality (ACM) endpoints. (B) Heart failure (HF) adverse event (AE) endpoints. The analysis was performed in patients with and without type 2 diabetes (T2D) or chronic kidney disease (CKD). For every endpoint included in the analysis, the overall effect was also reported. ^#^The effect is presented as a hazard ratio for empagliflozin versus placebo estimated with the use of a Cox proportional hazard model unless indicated otherwise. ^^^The effect is presented as the rate ratio estimated with the use of negative binomial regression analysis. *Adjusted number of events per 100 patient‐years (pt‐yrs), as calculated with the use of negative binomial regression analysis. Patients without T2D or CKD, *n* = 3489 (empagliflozin, *n* = 1753; placebo, *n* = 1736). Patients with T2D or CKD, *n* = 3033 (empagliflozin, *n* = 1507; placebo, *n* = 1526). CI, confidence interval.

The incidence rate in the placebo group in patients without and with T2D or CKD for a first event of HHF or all‐cause death was 4.02 and 9.65, for a first event of HHF was 2.19 and 4.80, and for a first event of all‐cause mortality was 2.15 and 5.62 events per 100 patient‐years. The adjusted event rate for total HHF events was 2.31 and 6.04 events per 100 patient‐years in study participants without and with an established recommendation for an SGLT2i. For the first AE of HF, including outpatient HF events, the incidence rate was 4.05 and 7.77 events per 100 patient‐years, whereas the adjusted event rate for total AEs of HF was 4.30 and 9.58 events per 100 patient‐years among individuals without and with T2D or CKD. For the first AE of HF or all‐cause mortality, the incidence rate was 5.67 and 11.95 events per 100 patient‐years, whereas the adjusted event rate for total AEs of HF and all‐cause mortality was 7.27 and 19.47 events per 100 patient‐years among individuals without and with T2D or CKD (*Figure* *1*).

### Outcomes

No differences between treatment groups were observed for time to first HHF or all‐cause death overall and in those without and with T2D or CKD [hazard ratio (HR) 0.93; 95% confidence interval (CI) 0.70, 1.23; and HR 0.88; 95% CI 0.72, 1.08, respectively; *P* for interaction = 0.77]. Empagliflozin reduced time to first HHF, total number of HHFs, time to first AE of HF (including outpatient HF events), total AEs of HF, time to first AE of HF or all‐cause death, and total number of AEs of HF or all‐cause death, overall and consistently in study participants without and with T2D or CKD (*Figure* *1*). Among individuals without T2D or CKD, a first event of HHF occurred in 41 and 54 patients in the empagliflozin and placebo groups (incidence rate 1.63 and 2.19 events per 100 patient‐years, respectively; HR 0.72; 95% CI 0.48, 1.08), while in those with T2D or CKD, it occurred in 77 and 99 in the empagliflozin and placebo groups (incidence rate 3.75 and 4.80 events per 100 patient‐years, respectively; HR 0.79; 95% CI 0.59, 1.07; *P* for interaction = 0.71). In study participants without T2D or CKD, the total number of HHFs occurred in 50 and 63 patients in the empagliflozin and placebo groups (adjusted event rate 1.74 and 2.31 events per 100 patient‐years, respectively; rate ratio (RR) 0.75; 95% CI 0.48, 1.18), while in those with T2D or CKD, it occurred in 98 and 144 patients in the empagliflozin and placebo groups (adjusted event rate 3.91 and 6.04 events per 100 patient‐years, respectively; RR 0.65; 95% CI 0.45, 0.94; *P* for interaction = 0.61).

**Figure 2:**
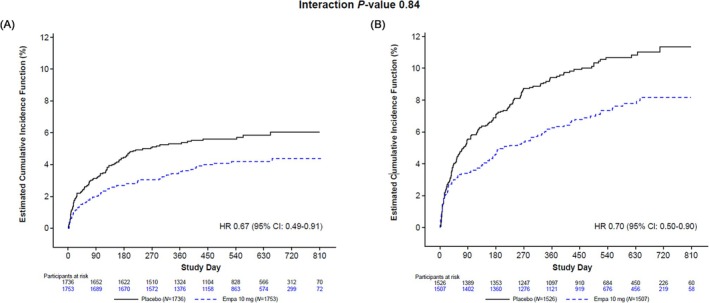
Time to first adverse event of heart failure. The analysis was performed in (A) absence and (B) presence of baseline estimated glomerular filtration rate <60 mL/min/1.73 m^2^ or history of chronic kidney disease or baseline type 2 diabetes. CI, confidence interval; Empa, empagliflozin; HR, hazard ratio.

**Figure 3:**
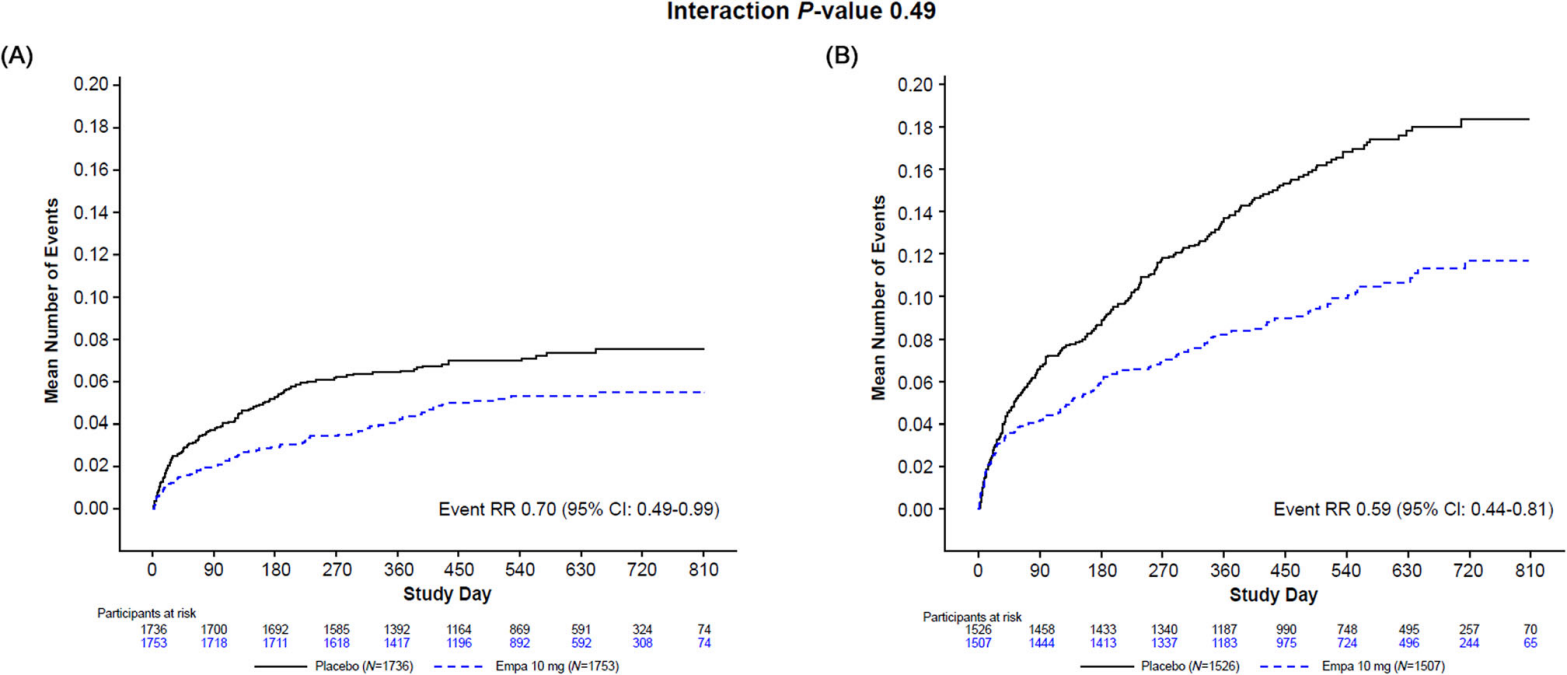
Total number of adverse events of heart failure. The analysis was performed in (A) absence and (B) presence of baseline estimated glomerular filtration rate <60 mL/min/1.73 m^2^ or history of chronic kidney disease or baseline type 2 diabetes. CI, confidence interval; Empa, empagliflozin; RR, rate ratio.

In study participants without T2D or CKD, a first AE of HF occurred in 70 and 98 patients in the empagliflozin and placebo groups (incidence rate 2.81 and 4.05 events per 100 patient‐years, respectively; HR 0.67; 95% CI 0.49, 0.91), while in those with T2D or CKD, it occurred in 108 and 156 patients in the empagliflozin and placebo groups (incidence rate 5.34 and 7.77 events per 100 patient‐years; HR 0.70; 95% CI 0.55, 0.90; *P* for interaction = 0.84; see the Graphical Abstract and *Figures* *1* and *2*). Similarly, in patients without T2D or CKD, the total number of AEs of HF occurred in 85 and 120 patients in the empagliflozin and placebo groups (adjusted event rate 3.01 and 4.30 events per 100 patient‐years, respectively; RR 0.70; 95% CI 0.49, 0.99), while in those with T2D or CKD, it occurred in 142 and 234 patients in the empagliflozin and placebo groups (adjusted event rate 5.68 and 9.58 events per 100 patient‐years, respectively; RR 0.59; 95% CI 0.44, 0.81; *P* for interaction = 0.49; *Figures* *1* and *3*). Subgroup analyses of efficacy endpoints by patients with and without T2D or CKD are shown in the Graphical Abstract and *Figure* *1*.

### Safety

In study participants without T2D or CKD, AEs occurred in 24.7% in the empagliflozin group and 23.8% in the placebo group (incidence rate 21.85 and 21.20 events per 100 patient‐years, respectively), while in those with T2D or CKD, AEs occurred in 30.9% in the empagliflozin group and 31.5% in the placebo group (incidence rate 29.86 and 30.57 events per 100 patient‐years, respectively). In patients without T2D or CKD, serious AEs occurred in 21.1% in the empagliflozin group and 21.4% in the placebo group (incidence rate 18.39 and 18.87 events per 100 patient‐years, respectively), while in those with T2D or CKD, it occurred in 26.6% in the empagliflozin group and 28.5% in the placebo group (incidence rate 25.30 and 27.43 events per 100 patient‐years, respectively; *Table* *2*). Overall, the AE and serious AE rates were comparable between the treatment arms in both subgroups (without and with T2D or CKD).

**Table 2:** Safety data.

Event	Patients without T2D or CKD (*n* = 3489)				Patients with T2D or CKD (*n* = 3033)			
	Empagliflozin *n* = 1744		Placebo *n* = 1722		Empagliflozin *n* = 1490		Placebo *n* = 1507	
	*N* with event (%)	Incidence (per 100 patient‐years)	*N* with event (%)	Incidence (per 100 patient‐years)	*N* with event (%)	Incidence (per 100 patient‐years)	*N* with event (%)	Incidence (per 100 patient‐years)
Any adverse event	430 (24.7)	21.85	409 (23.8)	21.20	461 (30.9)	29.86	474 (31.5)	30.57
Adverse event leading to permanent treatment discontinuation	55 (3.2)	2.40	47 (2.7)	2.09	67 (4.5)	3.59	75 (5.0)	4.02
Serious adverse event	368 (21.1)	18.39	368 (21.4)	18.87	397 (26.6)	25.30	430 (28.5)	27.43
Ketoacidosis	0	0	0	0	2 (0.1)	0.11	1 (0.1)	0.05
Hepatic injury	6 (0.3)	0.26	1 (0.1)	0.04	2 (0.1)	0.11	1 (0.1)	0.05
Acute kidney injury^b^	7 (0.4)	0.30	13 (0.8)	0.58	20 (1.3)	1.07	30 (2.0)	1.62
Acute renal failure^a^	11 (0.6)	0.48	17 (1.0)	0.76	32 (2.1)	1.72	42 (2.8)	2.28
Urinary tract infection^c^	19 (1.1)	0.83	6 (0.3)	0.27	13 (0.9)	0.70	15 (1.0)	0.81
Volume depletion^c^	17 (1.0)	0.74	22 (1.3)	0.98	18 (1.2)	0.97	18 (1.2)	0.97
Hypoglycaemia^a^	0	0	0	0	4 (0.3)	0.21	5 (0.3)	0.27
Hypotension^c^	16 (0.9)	0.70	21 (1.2)	0.94	18 (1.2)	0.97	15 (1.0)	0.81

*Note*: Patients who received at least one dose of empagliflozin or placebo were included. Adverse events analysed up to 7 days after the discontinuation of the trial regimen are shown. Adverse events that were to be reported in the trial included serious adverse events, adverse events that led to discontinuation of the trial regimen for at least 7 days, and adverse events of special interest, defined as ketoacidosis, adverse events leading to lower‐limb amputation, hepatic injury and contrast‐induced kidney injury.

Abbreviations: CKD, chronic kidney disease; MedDRA, Medical Dictionary for Regulatory Activities; T2D, type 2 diabetes.

^a^ Events were identified with the use of a standardized MedDRA, Version 26.1, query.

^b^ ‘Acute kidney injury’ is a MedDRA, Version 26.1, preferred term.

^c^ Events were identified with the use of a Boehringer Ingelheim‐customized MedDRA, Version 26.1, query.

## Discussion

Whereas empagliflozin did not reduce the primary endpoint of HHF or all‐cause death in the EMPACT‐MI trial,^11^ pre‐specified analyses demonstrated a favourable impact on HF outcomes, reducing first HHF and the total number of HHF. Benefits of empagliflozin were also seen in exploratory analyses for risk reduction in HF AEs, including outpatient HF events as well as across the range of LVEF.^10^, ^17^ In the current analysis, these effects were seen in study participants with and without T2D or CKD. Our results confirm the consistency in the efficacy and safety of empagliflozin after an acute MI among patients without and with an established recommendation for an SGLT2i. This is also important because our data confirm the safety of starting empagliflozin in an acute MI setting in those with an established recommendation for an SGLT2i, concurrently providing a benefit for the primary condition (i.e., T2D or CKD) including a reduction in HF outcomes. In fact, we confirmed the well‐established safety profile of empagliflozin, initiated within the first 2 weeks of an acute MI, which was consistent in both cohorts. Similar rates for any AEs and serious AEs were seen for empagliflozin and placebo arms across patients with and without T2D or CKD. These results highlight the potential role of empagliflozin in reducing HF after MI regardless of having an established recommendation for an SGLT2i, reassuring early safe post‐MI initiation of empagliflozin in those with T2D or CKD and highlighting HF‐related benefit in those without these conditions as well.

Clinical practice guidelines emphasize the early detection of individuals at risk for developing HF after AMI,^12^, ^13^, ^18^, ^19^ a circumstance that is associated with a worse prognosis.^3^ In particular, individuals in whom LV dysfunction persists after an ischaemic event are at increased risk of developing chronic HF, death and hospitalizations.^1^, ^3^, ^4^ Despite advances in interventional and medical therapies targeting recurrent MI, no new therapy has been discovered to reduce the risk of new‐onset HF after MI in two decades. In recent years, SGLT2is have shown to reduce the risk of HF events across various patient populations, in particular in those at high risk of developing HF (including those with T2D and CKD) and in patients with prevalent HF, irrespective of LVEF.^7^, ^8^, ^9^ Recently, analyses of the EMPACT‐MI trial showed that empagliflozin may have a role for the treatment of patients after AMI who are at risk for HF by reducing the risk for HF events.^10^ The current post hoc results extend this finding by demonstrating consistency in HF effect and safety of empagliflozin after MI regardless of T2D or CKD. In the present analysis, the event rates within the placebo group were more pronounced in those with T2D or CKD. This confirms that patients with established recommendations for an SGLT2i (such as T2D and/or CKD) are at higher risk of events compared with those without these conditions. Thus, patients admitted for MI with T2D and/or CKD should be treated with SGLT2i soon after the index event in order to provide an early prognostic benefit. Further, in‐hospital initiation of drugs may be associated with better adherence compared with outpatient initiation.^20^, ^21^

Our analysis confirmed the well‐established safety profile of empagliflozin in the AMI setting. There were no differences in overall AEs and serious AEs between empagliflozin and placebo in both patients with and without T2D or CKD. More AEs and serious AEs in the group with T2D or CKD were observed in both treatment groups, probably as a consequence of the higher comorbidity burden of patients affected by these conditions.

### Limitations

Our study should be considered within the context of its limitations. The EMPACT‐MI trial focused on clinical outcomes and did not collect mechanistic data. The trial focused on randomizing patients early after MI, a time in which patients can have stunned myocardium that may recover especially after revascularization and independent of concomitant pharmacotherapy.^2^ Furthermore, patients in the CKD trials could also participate with eGFR 60–90 mL/min/1.73 m^2^ and albuminuria; as such, they can be considered to have an established recommendation for SGLT2i as well.^8^ Because data regarding albuminuria were not collected in the EMPACT‐MI trial, these patients were not specifically evaluated in our post hoc analysis. However, we considered ‘history of CKD’ as an established recommendation for an SGLT2i, thus also including those with albuminuria and an eGFR ≥ 60 mL/min/1.73 m^2^. The outcomes of HF hospitalizations were not centrally adjudicated and were assessed by site investigators according to pre‐specified definitions with collection of corresponding data in structured electronic case report forms and evaluated through monitoring for completeness. However, we got robust findings regardless of how HF was assessed. Further efforts are needed to recruit more women and non‐white people in future randomized clinical trials given their lack of representativeness in large outcome studies impacting clinical practice.^22^

In conclusion, the effect of empagliflozin on first and total HHF and AEs of HF outcomes following AMI was consistent for individuals with and without T2D or CKD: These data suggest the potential role for empagliflozin in preventing HF events in patients after MI, as well as a lack of heterogeneity in treatment effect among those with and without pre‐existing T2D or CKD.

## Conflict of interest statement

The authors meet criteria for authorship as recommended by the ICMJE. The authors did not receive payment related to the development of the manuscript. Boehringer Ingelheim was given the opportunity to review the manuscript for medical and scientific accuracy as well as intellectual property considerations. F. Fioretti has nothing to declare. J. Butler has served as a consultant to Abbott, American Regent, Amgen, Applied Therapeutics, AskBio, Astellas, AstraZeneca, Bayer, Boehringer Ingelheim, Boston Scientific, Bristol Myers Squibb, Cardiac Dimensions, Cardiocell, Cardior, CSL Behring, CVRx, Cytokinetics, Daxor, Edwards, Element Science, Faraday, Foundry, G3P, Innolife, Impulse Dynamics, Imbria, Inventiva, Ionis, Lexicon, Lilly, LivaNova, Janssen, Medtronic, Merck, Occlutech, Owkin, Novartis, Novo Nordisk, Pfizer, Pharmacosmos, PharmaIN, Prolaio, Regeneron, Renibus, Roche, Salamandra, Sanofi, SC Pharma, Secretome, Sequana, SQ Innovation, Tenex, Tricog, Ultromics, Vifor and ZOLL. J. A. Udell reports personal fees from Amgen, AstraZeneca, Eli Lilly and Company, Novavax, Novo Nordisk and Sanofi; grants and personal fees from Boehringer Ingelheim; and grants from Novartis. W. Schuyler Jones reports research grants from Bayer, Boehringer Ingelheim, Merck, Novartis, PCORI and the National Institutes of Health. M. C. Petrie reports research funding from Boehringer Ingelheim, Roche, SQ Innovations, AstraZeneca, Novartis, Novo Nordisk, Medtronic, Boston Scientific and Pharmacosmos and consultancy or trial committee participation from Akero, Applied Therapeutics, Amgen, AnaCardio, Biosensors, Boehringer Ingelheim, Novartis, AstraZeneca, Novo Nordisk, AbbVie, Bayer, Horizon Therapeutics, Takeda, Cardiorentis, Pharmacosmos, Siemens, Eli Lilly and Company, Vifor, New Amsterdam, Moderna, Teikoku, LIB Therapeutics and 3R Life Sciences. J. Harrington receives salary support from T32 Training Grant T32HL069749. M. Mattheus is an employee of Boehringer Ingelheim. J. Bauersachs received honoraria for lectures/consulting from Novartis, Vifor, Bayer, Pfizer, Boehringer Ingelheim, AstraZeneca, Cardior, CVRx, BMS, Amgen, Corvia, Norgine, Edwards and Roche not related to this article and research support for Dr Bauersachs's department from ZOLL, CVRx, Abiomed, Norgine and Roche not related to this article. A. Bayes‐Genis received honoraria for lectures/consulting from Abbott, Novartis, Vifor, Bayer, Boehringer Ingelheim, AstraZeneca, Medtronic and Roche not related to this article. S. G. Goodman reports research grant support (e.g., steering committee or data and safety monitoring committee) or speaker or consulting honoraria (e.g., advisory boards) from Amgen, Anthos Therapeutics, AstraZeneca, Bayer, Boehringer Ingelheim, Bristol Myers Squibb, CSL Behring, CYTE Ltd, Daiichi Sankyo/American Regent, Eli Lilly and Company, Esperion, Ferring Pharmaceuticals, HLS Therapeutics, Idorsia, JAMP Pharma, Merck, Novartis, Novo Nordisk A/C, Pendopharm/Pharmascience, Pfizer, Regeneron, Sanofi, Servier, Tolmar Pharmaceuticals and Valeo Pharma and salary support or honoraria from the Heart and Stroke Foundation of Ontario/University of Toronto (Polo) Chair, Canadian Heart Failure Society, Canadian Heart Research Centre and MD Primer, Canadian VIGOUR Centre, Cleveland Clinic Coordinating Center for Clinical Research, Duke Clinical Research Institute, New York University Clinical Coordinating Center, PERFUSE Research Institute and TIMI Study Group (Brigham Health). T. Gasior is an employee of Boehringer Ingelheim. J. L. Januzzi reports participation as a board member of Imbria Pharmaceuticals and director at Jana Care; has received research support from Abbott, Applied Therapeutics, Bayer, BBMS, HeartFlow Inc., Innolife and Roche Diagnostics and consulting income from Abbott, AstraZeneca, Bayer, Beckman, Boehringer Ingelheim, Janssen, Medtronic, Novartis, Prevencio, QuidelOrtho, Roche Diagnostics and Vascular Dynamics; and participates in clinical endpoint committees or data safety monitoring boards for Abbott, AbbVie, Bayer, CVRx, Medtronic, Pfizer, Roche Diagnostics and Takeda. R. D. Lopes reports research grants or contracts from Amgen, Bristol Myers Squibb, GlaxoSmithKline, Medtronic, Pfizer and Sanofi‐Aventis; funding for educational activities or lectures from Pfizer, Daiichi Sankyo and Novo Nordisk; and funding for consulting or other services from Bayer, Boehringer Ingelheim, Bristol Myers Squibb and Novo Nordisk. P. Ponikowski received consultancy fees and speaker's honoraria from Boehringer Ingelheim, AstraZeneca, Vifor Pharma, Servier, Novartis, Bayer, MSD, Abbott Vascular, Pharmacosmos, Relaxera, Novo Nordisk and Radcliffe Group. X. Rossello has been a member of the steering committee of the EMPACT‐MI through a CVCT fellowship, though he has not received any honorarium for this task and has nothing else to declare. M. Schou reports lecture fees from Novartis, AstraZeneca, Boehringer Ingelheim and Novo Nordisk. P. van der Meer reports support from the European Research Council (ERC CoG 101045236, DISSECT‐HF); the UMCG, which employs Dr van der Meer, received consultancy fees or grants from Novartis, Pharmacosmos, Vifor Pharma, AstraZeneca, Pfizer, Pharma Nord, BridgeBio, Novo Nordisk, Daiichi Sankyo, Boehringer Ingelheim and Ionis. D. Vinereanu reports research grants and consultancy fees from Boehringer Ingelheim and research grants from Bayer Healthcare, Novartis and Servier Pharmaceuticals LLC. S. Zieroth received research grant support, served on advisory boards for or had speaker engagements with Abbott, AstraZeneca, Bayer, BMS, Boehringer Ingelheim, CSL Vifor, Cytokinetics, Edwards, Eli Lilly and Company, GSK, Medtronic, Merck, Novartis, Novo Nordisk and Pfizer and serves on a clinical trial committee for studies sponsored by AstraZeneca, Boehringer Ingelheim, Cytokinetics, Merck, Novartis, Pfizer and Salubris Bio. Non‐industry relationships include involvement with the Canadian Medical & Surgical Knowledge Translation Research Group, CCS, CHFS, Charité, EOCI, Liv, Medscape, Ology, PACE‐CME, Radcliffe Group, Reach MD, Translational Medicine Academy, and Voxmedia. M. Brueckmann is an employee of Boehringer Ingelheim. M. Sumin is an employee of Boehringer Ingelheim. D. L. Bhatt is a member of the Advisory Board of Angiowave, Bayer, Boehringer Ingelheim, CellProthera, Cereno Scientific, Elsevier Practice Update Cardiology, High Enroll, Janssen, Level Ex, McKinsey, Medscape Cardiology, Merck, MyoKardia, NirvaMed, Novo Nordisk, PhaseBio, PLx Pharma, and Stasys. He serves on the Board of Directors for the American Heart Association New York City, Angiowave (stock options), Bristol Myers Squibb (stock), DRS. LINQ (stock options), and High Enroll (stock). He has served as a consultant for Broadview Ventures, GlaxoSmithKline, Hims, SFJ, and Youngene. He has participated on Data Monitoring Committees for Acesion Pharma, Assistance Publique–Hôpitaux de Paris, Baim Institute for Clinical Research (formerly Harvard Clinical Research Institute, for the PORTICO trial funded by St. Jude Medical, now Abbott), Boston Scientific (Chair, PEITHO trial), Cleveland Clinic, Contego Medical (Chair, PERFORMANCE 2), Duke Clinical Research Institute, Mayo Clinic, Mount Sinai School of Medicine (for the ENVISAGE trial funded by Daiichi Sankyo; for the ABILITY‐DM trial funded by Concept Medical; and for ALLAY‐HF funded by Alleviant Medical), Novartis, Population Health Research Institute, and Rutgers University (for the NIH‐funded MINT trial). He has received honoraria from the American College of Cardiology (Senior Associate Editor, Clinical Trials and News, ACC.org; Chair, ACC Accreditation Oversight Committee), Arnold and Porter law firm (related to Sanofi/Bristol Myers Squibb clopidogrel litigation), Baim Institute for Clinical Research (for RE‐DUAL PCI clinical trial steering committee funded by Boehringer Ingelheim and AEGIS‐II executive committee funded by CSL Behring), Belvoir Publications (Editor in Chief, Harvard Heart Letter), Canadian Medical and Surgical Knowledge Translation Research Group (clinical trial steering committees), CSL Behring (AHA lecture), Cowen and Company, Duke Clinical Research Institute (clinical trial steering committees, including PRONOUNCE trial funded by Ferring Pharmaceuticals), HMP Global (Editor in Chief, Journal of Invasive Cardiology), Journal of the American College of Cardiology (Guest Editor; Associate Editor), K2P (Co‐Chair, interdisciplinary curriculum), Level Ex, Medtelligence/ReachMD (CME steering committees), MJH Life Sciences, Oakstone CME (Course Director, Comprehensive Review of Interventional Cardiology), Piper Sandler, Population Health Research Institute (for the COMPASS operations committee, publications committee, steering committee, and USA national co‐leader, funded by Bayer), WebMD (CME steering committees), and Wiley (steering committee). He is Deputy Editor for Clinical Cardiology. He is named on a patent for sotagliflozin, assigned to Brigham and Women’s Hospital and subsequently assigned to Lexicon, although neither he nor Brigham and Women’s Hospital receive any income from this patent. He has received research funding from Abbott, Acesion Pharma, Afimmune, Aker Biomarine, Alnylam, Amarin, Amgen, AstraZeneca, Bayer, Beren, Boehringer Ingelheim, Boston Scientific, Bristol Myers Squibb, Cardax, CellProthera, Cereno Scientific, Chiesi, CinCor, Cleerly, CSL Behring, Eisai, Ethicon, Faraday Pharmaceuticals, Ferring Pharmaceuticals, Forest Laboratories, Fractyl, Garmin, HLS Therapeutics, Idorsia, Ironwood, Ischemix, Janssen, Javelin, Lexicon, Lilly, Medtronic, Merck, Moderna, MyoKardia, NirvaMed, Novartis, Novo Nordisk, Otsuka, Owkin, Pfizer, PhaseBio, PLx Pharma, Recardio, Regeneron, Reid Hoffman Foundation, Roche, Sanofi, Stasys, Synaptic, The Medicines Company, Youngene, and 89Bio. He receives royalties from Elsevier as Editor of Braunwald’s Heart Disease. He has served as Site Co‐Investigator for trials sponsored by Abbott, Biotronik, Boston Scientific, CSI, Endotronix, St. Jude Medical (now Abbott), Philips, SpectraWAVE, Svelte, and Vascular Solutions. He is a Trustee of the American College of Cardiology. He is also involved in unfunded research with FlowCo. A. F. Hernandez has served as a consultant for Amgen, AstraZeneca, Bayer, Boehringer Ingelheim, Boston Scientific, Bristol Myers Squibb, Cytokinetics, Eidos, GlaxoSmithKline, Intellia, Intercept, MyoKardia, Novartis, Novo Nordisk, Prolaio and TikkunLev and has received research funding from American Regent, Amgen, Bayer, Boehringer Ingelheim, Lilly, Merck, Novartis, Novo Nordisk and Verily. S. D. Anker receives grants and personal fees from Vifor and Abbott Laboratories and personal fees for consultancies, trial committee work and/or lectures from Actimed, AstraZeneca, Bayer, BioVentrix, Boehringer Ingelheim, Brahms, Cardiac Dimensions, Cardior, Cordio, CVRx, Cytokinetics, Edwards, Faraday Pharmaceuticals, GSK, HeartKinetics, Impulse Dynamics, Medtronic, Novartis, Novo Nordisk, Occlutech, Pfizer, Regeneron, Relaxera, Repairon, SCIRENT, Sensible Medical, Servier, Vectorious and V‐Wave. Co‐inventor on two patent applications related to MR‐proANP (DE 102007010834 and DE 102007022367); however, he receives no personal financial benefit from the issued patents.

## Funding

The study was supported and funded by Boehringer Ingelheim and Eli Lilly and Company Diabetes Alliance.

## Supporting information


**Table S1.** Index hospitalization details by patients with and without T2D/CKD. CKD = chronic kidney disease, eGFR = estimated glomerular filtration rate, IV = intravenous, LVEF = left ventricular ejection fraction, NA = not applicable, NSTEMI = non‐ST elevation myocardial infarction, STEMI = ST elevation myocardial infarction, T2D = type 2 diabetes mellitus. *Number of patients with missing data for type of myocardial infarction: in “subgroup without” N = 1 in empagliflozin and N = 0 in placebo, in “subgroup with” N = 1 in empagliflozin and N = 0 in placebo; for signs or symptoms of congestion with lowest LVEF < or ≥ 45%: in “subgroup without” N = 13 in empagliflozin and N = 14 in placebo, in “subgroup with” N = 10 in empagliflozin and N = 14 in placebo. ^§^Analysis conducted with pooled Empa and placebo; chi−square test for categorical variables.
**Table S2.** Enrichment criteria and risk factors for heart failure in patients with and without T2D/CKD. *Number of patients with missing data for LVEF <35%: in “subgroup without” N = 27, in “subgroup with” N = 25. ^#^Persistent or permanent atrial fibrillation, or paroxysmal if associated with index MI. ^^^based on log−transformed results. ^1^t‐test for continuous variables, chi−square test for categorical variables. Except for eGFR, laboratory values and pulmonary artery pressure have been optional to be reported beyond meeting the inclusion criterion of providing at least 1 enrichment criterion.
