# Supplementary material for: Empagliflozin after myocardial infarction with or without diabetes and chronic kidney disease: Insights from EMPACT‐MI
Source: ESC Heart Fail. 2025 Sep 14;12(6):3940–52. doi: 10.1002/ehf2.15393 (PMC12719805; doi:10.1002/ehf2.15393)
Supplement: Supplementary file 1 — Table S1. Index hospitalization details by patients with and without T2D/CKD. CKD = chronic kidney disease, eGFR = estimated glomerular filtration rate, IV = intravenous, LVEF = left ventricular ejection fraction, NA = not applicable, NSTEMI = non‐ST elevation myocardial infarction, STEMI = ST elevation myocardial infarction, T2D = type 2 diabetes mellitus. *Number of patients with missing data for type of myocardial infarction: in “subgroup without” N = 1 in empagliflozin and N = 0 in placebo, in “subgroup with” N = 1 in empagliflozin and N = 0 in placebo; for signs or symptoms of congestion with lowest LVEF < or ≥ 45%: in “subgroup without” N = 13 in empagliflozin and N = 14 in placebo, in “subgroup with” N = 10 in empagliflozin and N = 14 in placebo. §Analysis conducted with pooled Empa and placebo; chi−square test for categorical variables. Table S2. Enrichment criteria and risk factors for heart failure in patients with and without T2D/CKD. *Number of patients with missing data for LVEF <35%: in “subgroup without” N = 27, in “subgroup with” N = 25. #Persistent or permanent atrial fibrillation, or paroxysmal if associated with index MI. ^based on log−transformed results. 1t‐test for continuous variables, chi−square test for categorical variables. Except for eGFR, laboratory values and pulmonary artery pressure have been optional to be reported beyond meeting the inclusion criterion of providing at least 1 enrichment criterion. [file EHF2-12-3940-s001.docx]

**Table S1**. Index hospitalization details by patients with and without T2D/CKD.

|  | Patients **without** baseline eGFR<60 mL/min/1.73 m^2^ and history of CKD and baseline T2D (n=3489) | | | Patients **with** baseline eGFR<60 mL/min/1.73 m2 or history of CKD or baseline T2D (n= 3033) | | |  |
| --- | --- | --- | --- | --- | --- | --- | --- |
|  | Empagliflozin  n = 1753 | Placebo n =1736 | Total  n = 3489 | Empagliflozin  n = 1507 | Placebo n = 1526 | Total n = 3033 | P^§^ |
| ST elevation myocardial infarction, N (%) | 1387 (79.1) | 1336 (77.0) | 2723 (78.0) | 1057 (70.1) | 1065 (69.8) | 2122 (70.0) | <0.0001 |
| Non-ST elevation myocardial infarction, N (%) | 365 (20.8) | 400 (23.0) | 765 (21.9) | 449 (29.8) | 461 (30.2) | 910 (30.0) |  |
| Revascularization done, N (%) | 1601 (91.3) | 1589 (91.5) | 3190 (91.4) | 1310 (86.9) | 1322 (86.6) | 2632 (86.8) | <0.0001 |
| Fibrinolytic therapy given, N (%) | 200 (11.4) | 201 (11.6) | 401 (11.5) | 145 (9.6) | 154 (10.1) | 299 (9.9) | 0.03 |
| Lowest LVEF during admission (%) | 39±9 | 40±9 | 39±9 | 41±9 | 41±9 | 41±9 | <0.0001 |
| Signs or symptoms of congestion, N (%) | 1044 (59.6) | 990 (57.0) | 2034 (58.3) | 1027 (68.1) | 1084 (71.0) | 2111 (69.6) | <0.0001 |
| With lowest LVEF <45%* | 598 (34.1) | 547 (31.5) | 1145 (32.8) | 574 (38.1) | 604 (39.6) | 1178 (38.8) | // |
| With lowest LVEF >45%* | 298 (17.0) | 303 (17.5) | 601 (17.2) | 359 (23.8) | 381 (25.0) | 740 (24.4) |  |
| Treatment of congestion, N (%) | 909 (51.9) | 864 (49.8) | 1773 (50.8) | 943 (62.6) | 999 (65.5) | 1942 (64.0) | <0.0001 |
| Clinically significant acute kidney injury during admission prior to randomization, N (%) | 21 (1.2) | 14 (0.8) | 35 (1.0) | 73 (4.8) | 93 (6.1) | 166 (5.5) | <0.0001 |

CKD = chronic kidney disease, eGFR = estimated glomerular filtration rate, IV = intravenous, LVEF = left ventricular ejection fraction, NA = not applicable, NSTEMI = non-ST elevation myocardial infarction, STEMI = ST elevation myocardial infarction, T2D = type 2 diabetes mellitus.

*****Number of patients with missing data for type of myocardial infarction: in “subgroup without” N=1 in empagliflozin and N=0 in placebo, in “subgroup with” N=1 in empagliflozin and N=0 in placebo; for signs or symptoms of congestion with lowest LVEF < or > 45%: in “subgroup without” N= 13 in empagliflozin and N=14 in placebo, in “subgroup with” N=10 in empagliflozin and N=14 in placebo.

^§^Analysis conducted with pooled Empa and placebo; chi−square test for categorical variables.

**Table S2**. Enrichment criteria and risk factors for heart failure in patients with and without T2D/CKD.

|  | Patients without baseline eGFR<60 mL/min/1.73 m^2^ and history of CKD and baseline T2D (n=3489) | Patients with baseline eGFR<60 mL/min/1.73 m^2^ or history of CKD or baseline T2D (n= 3033) | P^1^ |
| --- | --- | --- | --- |
| Age > 65 years, N (%) | 1533 (43.9) | 1727 (56.9) | <0.0001 |
| Female, N (%) | 708 (20.3) | 917 (30.2) | <0.0001 |
| Left ventricular ejection fraction <35%*, N (%) | 968 (27.7) | 704 (23.2) | <0.0001 |
| Prior myocardial infarction, N (%) | 391 (11.2) | 456 (15.0) | <0.0001 |
| Atrial fibrillation^#^, N (%) | 307 (8.8) | 382 (12.6) | <0.0001 |
| Increased N-terminal pro-B type natriuretic peptide, N (%) | 1029 (29.5) | 825 (27.2) | <0.0001 |
| Baseline N-terminal pro-B type natriuretic peptide (pg/mL) | 2534.71 ± 3148.52 | 3673.45 ± 5008.09 | 0.0001^^^ |
| Pulmonary artery systolic pressure >40 mmHg, N (%) | 320 (9.2) | 278 (9.2) | 0.93 |
| No revascularization, N (%) | 232 (6.6) | 305 (10.1) | <0.0001 |
| 3−vessel coronary disease, N (%) | 1047 (30.0) | 976 (32.2) | 0.0002 |
| Number of enrichment factors, N (%) | | | |
| > 2 factors | 1918 (55.0) | 2680 (88.4) | <0.0001 |
| > 3 factors | 789 (22.6) | 1979 (65.2) | <0.0001 |
| > 4 factors | 240 (6.9) | 1182 (39.0) | <0.0001 |
| ST elevation myocardial infarction | 2723 (78.0) | 2122 (70.0) | <0.0001 |
| Non-ST elevation myocardial infarction | 765 (21.9) | 910 (30.0) |  |
| Symptoms or signs of congestion, N (%) | 2034 (58.3) | 2111 (69.6) | <0.0001 |
| Type 2 diabetes mellitus at baseline, N (%) | 0 | 2081 (68.6) | <0.0001 |
| No history of chronic kidney disease, N (%) | 3489 (100.0) | 2579 (85.0) | <0.0001 |
| Estimated glomerular filtration rate <60 mL/min/1.73m² during hospitalization, N (%) | 72 (2.1) | 1438 (47.4) | <0.0001 |

*Number of patients with missing data for LVEF <35%: in “subgroup without” N=27, in “subgroup with” N=25.
^#^Persistent or permanent atrial fibrillation, or paroxysmal if associated with index MI.
^^^based on log−transformed results.
^1^ t-test for continuous variables, chi−square test for categorical variables.

Except for eGFR, laboratory values and pulmonary artery pressure have been optional to be reported beyond meeting the inclusion criterion of providing at least 1 enrichment criterion.
